# Why the FUSS (Fentanyl Urine Screen Study)? A cross-sectional survey to characterize an emerging threat to people who use drugs in British Columbia, Canada

**DOI:** 10.1186/s12954-015-0088-4

**Published:** 2015-11-14

**Authors:** Ashraf Amlani, Geoff McKee, Noren Khamis, Geetha Raghukumar, Erica Tsang, Jane A. Buxton

**Affiliations:** British Columbia Centre for Disease Control, 655 West 12th Avenue, Vancouver, BC V5Z 4R4 Canada; Faculty of Medicine, University of British Columbia, Vancouver, Canada; School of Public and Population Health, University of British Columbia, Vancouver, Canada

**Keywords:** Survey, People who use drugs, Fentanyl, Overdose, Opioids

## Abstract

**Background:**

Fentanyl-detected illicit drug overdose deaths in British Columbia (BC) recently increased dramatically from 13 deaths in 2012 to 90 deaths in 2014, signaling an emerging public health concern. Illicit fentanyl is sold as pills or powders, often mixed with other substances like heroin or oxycodone; reports from coroners suggested that fentanyl was frequently taken unknowingly by people who use drugs. This study aimed to assess the prevalence and characteristics of fentanyl use among clients accessing harm reduction (HR) services in BC.

**Methods:**

Participants attending HR services at 17 sites across BC were invited to complete an anonymous questionnaire describing drugs they have used within the last 3 days and provide a urine sample to test for fentanyl. Data from eligible participants were analyzed using descriptive, bivariate, and multivariate statistical methods.

**Results:**

Surveys from 17 HR sites were received, resulting in analysis of responses from 242 eligible participants. Most participants used multiple substances (median = 3), with crystal meth (59 %) and heroin (52 %) use most frequently reported. Seventy participants (29 %) tested positive for fentanyl, 73 % of whom did not report using fentanyl. Controlling for age, gender, and health authority, reported use of fentanyl (odds ratio (OR) = 6.13, 95 % confidence interval (CI) = [2.52, 15.78], *p* < 0.001) and crystal methamphetamine (OR = 3.82, 95 % CI = [1.79, 8.63], *p* < 0.001) use were significantly associated with fentanyl detection.

**Conclusions:**

The proportion of those testing positive who did not report knowingly using fentanyl represents a considerable public health concern. The risk of overdose among this vulnerable population highlights the need for targeted HR strategies, such as increased accessibility to naloxone, overdose education, and urine screens.

## Background

Use of illicit fentanyl has emerged as a dangerous trend among people who use drugs in British Columbia (BC). Fentanyl-detected, illicit drug overdose deaths in BC have increased dramatically between 2012 and 2015 [1]. Reports from the BC Coroners Service suggest many of those testing positive for fentanyl were unknowingly using the substance [1]. The rapid increase in fentanyl-detected overdoses and risk of unintentional administration presents an emerging public health concern in BC.

Fentanyl is a synthetic opioid, far more potent than morphine and heroin, clinically used in anesthesia, and for management of chronic pain, pharmaceutical fentanyl is only available as transdermal patches in Canada [2].

Recently in Canada, illicit fentanyl has been sold as pills or powders, often mixed with other substances like heroin or oxycodone and, on many occasions, ingested unintentionally by people who use drugs due to undisclosured pill/powder contents [2]. Historically, nonpharmaceutical fentanyl and its analogs have been sold under various street names, while recent preparations, called “green jellies” or “street oxy,” have become available across Canada [2, 3].

Fentanyl overdose can lead to respiratory depression, followed by decreased mental status, brain damage, and death. The potency of fentanyl considerably increases the risk of overdose. This is particularly concerning with illicit fentanyl, as doses can be highly variable and people using may be opioid naïve. The pharmacodynamic properties of fentanyl metabolites may also lead to prolonged physiologic effects in the context of an overdose, similar to longer acting opioids [4]. This complicates the emergency response and may increase the risk of complications, as patients can re-narcotize following reversal with naloxone [5].

Several clusters of fentanyl-related overdose deaths have been reported in both Canada and the USA. Between 2002 and 2004, 112 fentanyl-detected deaths were reported in Ontario [6]. These deaths were associated with a variety of fentanyl formulations and co-administered substances; however, a particular pattern of illicit use was not reported. A large cluster of 1013 fentanyl-related deaths between 2005 and 2007 in six US states was found to be associated with illicit fentanyl mixed with heroin and cocaine [7]. Another fentanyl-associated cluster of illicit drug deaths was identified in 2013 in Rhode Island [8].

Between 2009 and 2014 in Canada, fentanyl was deemed a cause or contributory cause in at least 655 deaths and was detected in at least 1019 drug poisoning deaths [9]. In BC, fentanyl-detected overdose deaths increased sevenfold from 13 deaths in 2012 to 90 in 2014 [9]. This represented a considerable increase in the proportion of total fentanyl-detected illicit drug overdose deaths, from 5 % in 2012 to over 25 % in 2014.

In response to the emerging threat of fentanyl use and subsequent overdose to people who use drugs in BC, this study was developed to assess the prevalence and characteristics of fentanyl use among clients accessing harm reduction (HR) services in BC. Increased understanding of these patterns may lead to more effectively targeted harm reduction strategies, such as health promotion campaigns targeting unsafe drug use practices and take-home naloxone programs.

## Methods

The study used a cross-sectional design linking surveys of demographics and substance usage patterns with fentanyl urine tests. The Behavioural Ethics Review Boards at the University of BC and Interior Health Authority granted ethics approval.

### Study materials

The survey used questions adapted from an annual survey of clients at HR sites conducted by the BC Centre for Disease Control [10]. Study materials were reviewed with a group of five peers (people with experience using drugs) at the Vancouver Area Network of Drug Users (VANDU). Materials were also distributed to potential sites in order to check for suitability and acceptability of study design, as there was potential for urine testing to be perceived as a threatening activity.

Although renal clearance of fentanyl varies by age and dosage, the urine levels of norfentanyl, a fentanyl metabolite, typically become negligible within 3 days of administration [11]. The surveys collected information on substance use within 3 days prior to survey completion and urine collection, as this was deemed the optimal window to link urine norfentanyl detection with fentanyl use.

After consultation with medical facilities already testing patients’ urine for fentanyl, BNTX Rapid Response TM fentanyl urine strip tests at a detection level of 20 ng/ml norfentanyl were chosen as the method for measuring urine norfentanyl levels [12].

### Recruitment and survey distribution

In BC, supplies for safer sex and drug use (e.g., needles and condoms) are distributed by a network of over 200 HR sites, including public health units and community service organizations [10]. We estimated a total sample size of 385 respondents would allow us to report results at 95 % confidence level of +/− 5 % for an unknown population. Based on general population density, 4–10 sites in each of five regional health authorities were invited directly by the British Columbia Centre for Disease Control (BCCDC) or the regional HR coordinator to participate in the study. Questionnaires, participant refusal tracking sheet, urine test strips and instructions, and honorarium funds were mailed to each participating site. Site staff were responsible for recruiting clients, getting informed consent from participants, administering the questionnaire, testing the urine sample, and providing the $5 participant honorarium. Unique client ID labels were used to maintain anonymity and link the survey, urine sample, and urine strip result. Data were collected from February through March 2015.

The sample population included individuals who reported using substances within the preceding 3 days, were over 19 years of age, presented to a participating site during the study period, and were able to give verbal, informed consent.

### Data cleaning and analysis

Data was entered into a Microsoft Access database and imported into the R statistical software environment for cleaning and analysis. This was performed using R package (version 3.1.1) & R Studio (version 0.98.1062) with functions from the MASS package (version 7.3-40).

Participants less than 19 years of age, who reported no substance use within the previous 3 days or whose fentanyl urine screen results were unlinked with the associated surveys, were excluded from analysis. Substance use data was cleaned and recoded for analysis. For the substance use questions, blanks were assumed to indicate negative responses.

Descriptive statistical techniques were applied to demographic and substance use variables. To maintain anonymity due to small cell sizes, location data was aggregated to the health authority level.

Odds ratios were calculated using logistic regression for number and type of substances used, health authority, gender, age group, and history of overdose. Analysis of variance (ANOVA) with the chi-square test was run on age and health authority variables to determine their significance. Significant variables were then controlled for age, gender, and health authority in a multivariate regression model.

## Results

**Fig. 1:**
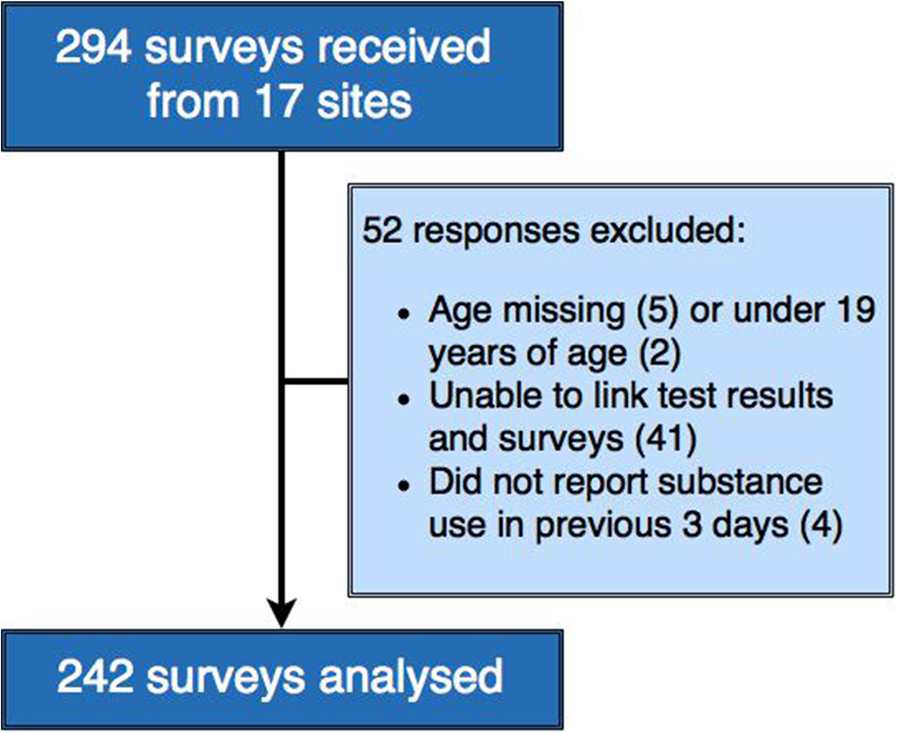
Outline of exclusion criteria for data analysis

Overall, 294 surveys were received from 17 HR supply distribution sites (Fig. 1). Only 28 individuals approached to participate in the study declined, corresponding to a participation rate of 91.3 %. Fifty-two participants were excluded from analysis resulting in a final sample size of 242:41 due to the absence of accurate linkage between urine test results and surveys, two were less than 19 years of age, five did not list their age, and four reported no substance use in the previous 3 days.

### Descriptive analysis

Demographic and substance use information is summarized in Table 1. A higher proportion of males (58 %) participated in the study than females (40 %), and 40–49-year-olds had the highest representation (34 %), followed by 30–39-years-olds (28 %). Sample sizes varied among the province’s five health authorities; Island Health was under-sampled and comprised only 6 % (*n* = 14) of total participants.

**Table 1:** Descriptive and bivariate analysis of demographics, substance use patterns, and associated fentanyl-detection as reported by participants

Variable	Total number (%)	Number positive (%)	OR [95 %CI]	*p* value
Substance use				
Methadone	73 (30 %)	18 (25 %)	0.74 [0.39, 1.36]	0.34
Morphine	97 (40 %)	27 (28 %)	0.91 [0.51, 1.61]	0.76
Dilaudid	55 (23 %)	13 (24 %)	0.71 [0.34, 1.39]	0.33
Oxycodone	23 (10 %)	9 (39 %)	1.67 [0.66, 4.00]	0.26
Fentanyl	31 (13 %)	19 (61 %)	4.97 [2.28, 11.20]	*<0.001*
Benzodiazapines	48 (20 %)	14 (29 %)	1.01 [0.49, 2.00]	0.97
Stimulants NOS^a^	25 (10 %)	7 (28 %)	0.95 [0.35, 2.30]	0.91
Heroin	126 (52 %)	42 (33 %)	1.57 [0.90, 2.78]	0.12
Cocaine powder	65 (27 %)	16 (25 %)	0.74 [0.38, 1.40]	0.37
Crack	78 (32 %)	21 (27 %)	0.86 [0.47, 1.56]	0.64
Crystal meth	143 (59 %)	55 (38 %)	3.50 [1.88, 6.86]	*<0.001*
Marijuana	55 (23 %)	14 (25 %)	0.80 [0.39, 1.55]	0.52
Number of substances used				
1 substance^b^	30 (12 %)	5 (17 %)	1.0	0.12
>1 substance	212 (88 %)	65 (31 %)	2.21 [0.87, 6.78]	0.12
Overdose				
Overdose within last month	24 (10 %)	7 (29 %)	0.98 [0.36, 2.39]	0.97
Overdose within last week	5 (2 %)	5 (100 %)	–	–
Health authority				
Fraser Health Authority	57 (24 %)	22 (39 %)	1.26 [0.59, 2.69]	<0.001
Interior Health Authority	54 (22 %)	9 (17 %)	0.40 [0.16, 0.96]	<0.001
Northern Health Authority	57 (24 %)	10 (18 %)	0.43 [0.17, 1.00]	<0.001
Vancouver Coastal Health^b^	60 (25 %)	20 (33 %)	- = 1.0	<0.001
Island Health	14 (6 %)	9 (64 %)	3.60 [1.10, 13.08]	<0.001
Age group				
19–29	45 (19 %)	12 (27 %)	0.99 [0.41, 2.31]	0.85
30–39^b^	67 (28 %)	18 (27 %)	1.0	0.85
40–49	83 (34 %)	27 (33 %)	1.31 [0.65, 2.70]	0.85
50+	47 (19 %)	13 (28 %)	1.04 [0.44, 2.40]	0.85
Gender				
Female	98 (40 %)	32 (33 %)	1.31 [0.75, 2.31]	0.34
Male^b^	141 (58 %)	38 (27 %)	1.0	0.34

^a^Not otherwise specified

^b^Variable used as reference for OR calculations

**Fig. 2:**
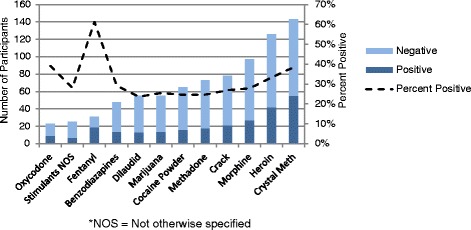
Prevalence of specific substance use and proportion of positive fentanyl test results among participants using these substances

**Fig. 3:**
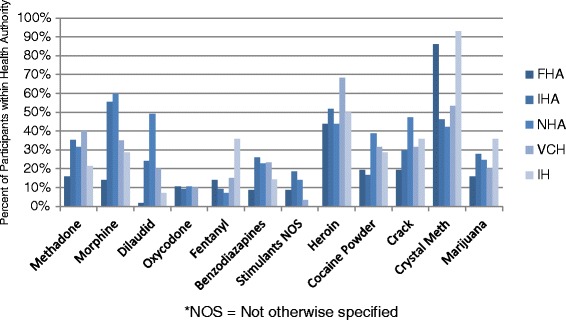
Percent of participants reporting specific substance use by health authority

As per Fig. 2, crystal methamphetamine was the most frequently reported substance used (58 %), followed by heroin (52 %). A total of 31 (13 %) individuals reported using fentanyl. Reported substance use varied significantly between health authorities (Fig. 3). The majority of participants reported using more than one substance (88 %) in the previous 3 days (median = 3). A total of 24 individuals (10 %) reported a history of overdose within the previous month and 5 (2 %) within the previous week. Approximately 29 % (*n* = 70) of the participants tested positive for fentanyl, 73 % (*n* = 51) of which reported no known fentanyl use within the previous 3 days. All participants reporting an overdose within the previous week (*n* = 5) tested positive for fentanyl, two of which did not report knowingly using the substance.

### Bivariate analysis

Odds ratios are summarized in Table 1. Among substance use patterns, those substances most significantly correlated with positive fentanyl urine screens were crystal methamphetamine (odds ratio (OR) = 3.50, 95 % confidence interval (CI) = [1.88, 6.86], *p* < 0.001) and fentanyl (OR = 4.97, 95 % CI = [2.28, 11.20], *p* < 0.001). Health authority was significantly correlated with a positive fentanyl urine screen (*p* < 0.01); in particular, Island Health was highly correlated (OR = 3.60, 95 % CI = [1.10, 13.08]). All other variables, including gender, age, history of overdose, and number of substances used, were found not to be significantly correlated with fentanyl detection.

### Multivariate analysis

A multivariate logistic regression model was created to control for associations between variables (Table 2). After controlling for age, gender, location, and health authority, fentanyl (OR = 6.13, 95 % CI = [2.52, 15.78], *p* <0.001) and crystal methamphetamine (OR = 3.82, 95 % CI = [1.79, 8.63], *p* <0.001) use remained significantly associated with fentanyl detection.

**Table 2:** Multivariate, additive logistic regression models of significant variables controlled for age, gender, location, and concurrent opioid use

Variable	OR [95 % CI]	*p* value
Fentanyl	6.13 [2.52, 15.78]	<0.001
Crystal meth	3.82 [1.79, 8.63]	<0.001

## Discussion

The patterns of substance use reported by participants differed from those identified by a recent provincial survey [13]. The higher reported use of crystal meth in our sample corresponds to anecdotal reports of a trend among experienced users in BC toward crystal methamphetamine and away from heroin and crack [14]. Differences in reported pattern of use between this study and the annual provincial survey may be due to smaller sample size (17 vs. 30+ sites) and time of year (winter vs. summer). BC’s natural geographic features and winter weather conditions may facilitate or restrict access to certain substances in various urban, rural, and remote communities. The significant correlation between location and fentanyl detection may be related to these factors.

The results of our study support the hypothesis that a considerable portion of fentanyl use in BC is unintentional, with 73 % of those testing positive for fentanyl, reporting no known fentanyl use within the previous 3 days. This represents a substantial risk to people who use drugs, as the dose of fentanyl in substances consumed may vary and individuals may be opioid naïve, creating an optimal scenario for overdose.

Reported use of heroin or other opioids besides fentanyl was not significantly correlated with fentanyl positivity, while crystal methamphetamine was significantly associated. The intentional inclusion of fentanyl in crystal methamphetamine by distributors is counter-intuitive, as opioids tend to oppose many of the intended effects of stimulants, and this group of users may be opioid naïve and more likely to overdose. Although this finding was unexpected, there have been reports of individuals using opioid/stimulant combinations, such as “speedballs” combining heroin and cocaine, as well as individuals experiencing opioid overdose symptoms following crystal methamphetamine use [15]. These combinations rely on the variability in pharmacodynamics between the opioid and stimulant, causing the transition between depressant and stimulant effects. Another possible explanation for the association may be unintentional contamination through handling and storage prior to distribution.

Intentional fentanyl use was highly correlated with fentanyl detection in the urine; however, 12 out of 31 participants reporting fentanyl use tested negative. It is possible that, due to the rapid clearance of fentanyl by the kidneys, fentanyl metabolites may be below the detectable level when a small amount and/or full 3 days between consumption and testing [12, 16]. Alternately, individuals reporting fentanyl use may actually be taking something entirely different.

The risk of overdose from unknown presence of fentanyl in street drugs highlights the need for strategies that focus on overdose prevention, recognition, and response. BC public health agencies have developed messaging campaigns to increase awareness of fentanyl-related overdoses and recommend precautionary strategies based on the unintentional fentanyl use identified by this study [17]. Providing overdose response training and naloxone, an opioid overdose antidote, is needed to reduce the harms of fentanyl overdose. Take-home naloxone programs implemented in BC and across North America support early reversal for opioid overdoses, as subsequent respiratory depression may lead to brain damage and death [18, 19]. While these programs traditionally target people who use opioids, the unintentional use of fentanyl may support a need to broaden coverage to people who use other substances, as well as their friends and family.

Given that illicit fentanyl may be mixed into many street drugs, the availability of street drug checking for fentanyl could reduce the risk of accidental overdose. However, in the absence of a cheap rapid fentanyl detection test that could be used on a drug sample, fentanyl urine testing strips could be provided to people who use drugs as an additional harm reduction service.

The elevated prevalence of fentanyl use also has implications on the use of naloxone by paramedics or in the emergency room (ER) to treat suspected opioid overdoses, and for the management of withdrawal for patients in detoxification centers. Given the pharmacodynamic properties of fentanyl, overdoses may result in more prolonged respiratory depression than other opioids and may require higher levels of naloxone for reversal [5]. Clinicians should consider fentanyl urine testing when managing overdoses in ER. Although this may not change the clinical management of the overdose, test results can serve as an opportunity to educate patients about their overdose and increase uptake of take-home naloxone programs.

In the time between the data collection phase and the publication of this study, fentanyl has become a public health concern in many jurisdictions outside BC, including other areas in Canada and the USA [9, 20, 21]. The recommendations stemming from our findings could reduce harms due to overdose in all regions affected by the presence of illicit fentanyl in the street market.

Several characteristics of this study limit the scope of our conclusions. Those sampled from participating HR sites are a small subset of individuals accessing harm reduction sites and of all people who use drugs in BC. The exclusion of 48 participants, in part due to the submission of unlinked surveys and test results from one site, also reduced the power of the study. Another site only sent samples and surveys of participants who tested positive for fentanyl, which may lead to an over estimate of fentanyl prevalence.

Technical limitations include cross-reactivity of the fentanyl urine tests and fentanyl analogs, such as sufentanyl. The rapid clearance of fentanyl may also result in negative test results, even if it was used within the 3-day window. The study was also limited by the exclusion of those younger than 19 years of age due to ethics approval; however, only a few of fentanyl-detected illicit drug deaths in BC have occurred in people under 19 years. Other limitations include reliance on self-reported questionnaires, the assumption that nonresponses represented negative answers and other biases inherent in cross-sectional study designs.

## Conclusions

The results of this study demonstrate that illicit fentanyl is a considerable risk to people who use drugs in BC, particularly among those who consume it unknowingly. The widespread use of crystal methamphetamine and its association with fentanyl detection suggests that even those using stimulants may be at risk of opioid overdose, thus emphasizing the importance of broadening overdose education and prevention programs.

Further research is required to verify the associations made in this report and may include investigation into drug distribution patterns, as well as a review of coroners’ files to identify substances implicated in illicit drug-associated deaths.

While the increase in fentanyl availability and fentanyl-detected deaths is alarming, support of harm reduction strategies can help mitigate the risks. Public health agencies have taken steps to combat this trend; however, further engagement is necessary to reduce the impact of illicit fentanyl on this vulnerable population.
